# Slow-wave sleep dysfunction in mild parkinsonism is associated with excessive beta and reduced delta oscillations in motor cortex

**DOI:** 10.3389/fnins.2024.1338624

**Published:** 2024-02-21

**Authors:** Ajay K. Verma, Bharadwaj Nandakumar, Kit Acedillo, Ying Yu, Ethan Marshall, David Schneck, Mark Fiecas, Jing Wang, Colum D. MacKinnon, Michael J. Howell, Jerrold L. Vitek, Luke A. Johnson

**Affiliations:** ^1^Department of Neurology, University of Minnesota, Minneapolis, MN, United States; ^2^Masonic Institute for the Developing Brain, University of Minnesota, Minneapolis, MN, United States; ^3^Division of Biostatistics, University of Minnesota, Minneapolis, MN, United States

**Keywords:** Parkinson’s disease, sleep-wake disturbances, MPTP, beta oscillations, delta oscillations, motor cortex, local field potential

## Abstract

Increasing evidence suggests slow-wave sleep (SWS) dysfunction in Parkinson’s disease (PD) is associated with faster disease progression, cognitive impairment, and excessive daytime sleepiness. Beta oscillations (8–35 Hz) in the basal ganglia thalamocortical (BGTC) network are thought to play a role in the development of cardinal motor signs of PD. The role cortical beta oscillations play in SWS dysfunction in the early stage of parkinsonism is not understood, however. To address this question, we used a within-subject design in a nonhuman primate (NHP) model of PD to record local field potentials from the primary motor cortex (MC) during sleep across normal and mild parkinsonian states. The MC is a critical node in the BGTC network, exhibits pathological oscillations with depletion in dopamine tone, and displays high amplitude slow oscillations during SWS. The MC is therefore an appropriate recording site to understand the neurophysiology of SWS dysfunction in parkinsonism. We observed a reduction in SWS quantity (*p* = 0.027) in the parkinsonian state compared to normal. The cortical delta (0.5–3 Hz) power was reduced (*p* = 0.038) whereas beta (8–35 Hz) power was elevated (*p* = 0.001) during SWS in the parkinsonian state compared to normal. Furthermore, SWS quantity positively correlated with delta power (*r* = 0.43, *p* = 0.037) and negatively correlated with beta power (*r* = −0.65, *p* < 0.001). Our findings support excessive beta oscillations as a mechanism for SWS dysfunction in mild parkinsonism and could inform the development of neuromodulation therapies for enhancing SWS in people with PD.

## Introduction

Slow-wave sleep (SWS), the deepest stage of non-rapid eye movement (NREM) sleep, plays a crucial role in learning and memory consolidation (Tononi and Cirelli, 2006; Léger et al., 2018). Dysfunction of SWS in people with advanced Parkinson’s disease (PD) is associated with faster disease progression (Schreiner et al., 2019), cognitive impairment (Schreiner et al., 2021; Wood et al., 2021), and daytime sleepiness (Schreiner et al., 2023), and is a major factor impacting the quality of life of people with PD. Whether SWS is also disrupted (reduction in SWS quantity) in the early disease state (i.e., mild parkinsonism) and the potential neural mechanism(s) driving dysfunction of SWS in mild parkinsonism remain unclear. An improved understanding of the pathophysiological changes associated with SWS dysfunction in mild parkinsonism can inform the development of targeted therapies to improve SWS in early PD and potentially slow disease progression (Morawska et al., 2021).

Excessive beta (8–35 Hz) oscillations in the basal ganglia-cortical circuit are considered by many to represent a neural biomarker associated with PD motor signs and are increasingly used to inform closed-loop deep brain stimulation (DBS) approaches (Swann et al., 2018; Little and Brown, 2020). Recent evidence suggests that beta oscillations may also play a role in sleep-wake disturbances in advanced parkinsonism (Hackius et al., 2016; Mizrahi-Kliger et al., 2020; Verma et al., 2022, 2023; Yin et al., 2023). Several studies have also shown that cortical delta oscillations, a hallmark of SWS, are reduced in an advanced stage of parkinsonism during SWS (Schreiner et al., 2021; Wood et al., 2021). Together these findings provide compelling support for investigating if the power of cortical delta and beta oscillations is altered in mild parkinsonism and whether they underlie SWS dysfunction. Based on these studies, we hypothesized that impairment in SWS will be associated with excessive cortical beta oscillations during SWS. We further hypothesize that excessive cortical beta oscillations during SWS will hinder the sustainment of delta oscillations in the cortex, a key feature of deep NREM sleep.

To elucidate the effect of mild parkinsonism on SWS quantity (% of recording time) and cortical neural oscillations in delta (0.5–3 Hz) and beta (8–35 Hz) bands, we recorded local field potentials (LFPs) from the primary motor cortex (MC) in a nonhuman primate (NHP) across normal and mildly parkinsonian sleep. The MC is a critical node in the basal ganglia thalamocortical (BGTC) network (Galvan et al., 2015) and can exhibit pathological oscillations with depletion in dopamine tone (Devergnas et al., 2014; Escobar Sanabria et al., 2017; Yu et al., 2021). Furthermore, MC displays high amplitude slow oscillations during SWS (Xu et al., 2019), a neural characteristic of SWS, hence a relevant recording site to understand the pathophysiology of SWS dysfunction associated with parkinsonism. The goal of this study was to provide insight regarding alterations in cortical neural oscillations as they relate to SWS dysfunction in an early stage of parkinsonism. The findings of this study will be critical for informing the development of targeted neurostimulation therapies to enhance neural oscillations in the cortex that can increase SWS quantity while suppressing neural oscillations detrimental to SWS.

## Methods

### Experimental protocol and data collection

All procedures were approved by the University of Minnesota Institutional Animal Care and Use Committee and complied with the US Public Health Service policy on the humane care and use of laboratory animals. One adult female rhesus macaque NHP (22 years old) was used in this study. The subject was instrumented with a 96-channel Microdrive (Gray Matter Research) with microelectrodes targeting basal ganglia, motor thalamus, and motor cortices. A subset of microelectrode channels (*n* = 9) in the MC that were not moved during normal and mild parkinsonian states were used for characterizing the effect of mild parkinsonism on SWS neurophysiology.

The video and wireless local field potential recording from MC during sleep-wake behavior were obtained while the subject was in its home enclosure using a Triangle BioSystem International (TBSI) and Tucker Davis Technology (TDT) recording systems across the normal and parkinsonian state at a sampling rate of ~24,000 Hz. The subject was rendered mildly parkinsonian by administering four weekly low-dose (0.2–0.3 mg/Kg) intramuscular injections of the neurotoxin 1-methyl-4-phenyl-1,2,3,6-tetrahydropyridine (MPTP). The MPTP administration was performed by experienced research staff as per the protocol established by our research center and adapted from Masilamoni et al. (2011) and Masilamoni and Smith (2018). After the 4th MPTP injection, the subject demonstrated mild motor impairment. After this point, the subject’s motor assessment was routinely monitored for stability for one month, after which the sleep recordings were performed. The severity of the subject’s parkinsonism on the side contralateral to the electrode implants was assessed using the modified version of the Unified Parkinson’s Disease Rating Scale (mUPDRS), which rates symptoms of bradykinesia, akinesia, rigidity, and tremor of the upper and lower limbs as well as food retrieval on a scale of 0–3 (0 = normal, 1 = mild, 2 = moderate, and 3 = severe), maximum score = 27 (adapted from Wang et al., 2022). A composite score of 3–9 was considered mild, 10–18 moderate, and 19–27 a severe parkinsonian state. The results presented in the manuscript are from 23 sessions of sleep recordings [6 normal and 17 mildly parkinsonian, mUPDRS = 4.70 (3.60–5.70), median (IQR)]. The sleep recordings began at approximately 7 pm (lights off at 6 p.m.) and resulted in 7.660 (7.331–7.664) hours [median (IQR)] of recording across normal and parkinsonian states presented in this study.

### Data analysis

Neural activities recorded from 9 adjacent microelectrode channels were averaged to derive MC LFP. The resultant MC LFP was bandpass filtered from 0.5 to 700 Hz then resampled to ~200 Hz and normalized to have unit standard deviation before further processing. Using the Welch power spectral density (PSD) the median power of MC delta oscillations (0.5–3 Hz) was computed on a second-by-second basis with a window size of 128 samples, 50% overlap, and 512 FFTs resulting in a frequency resolution of 0.39 Hz. The epochs were identified as slow wave sleep if the epoch was free from movement (determined using electromyography) and the power of MC delta oscillations was greater than four times the power of MC delta oscillations during the wake. The threshold for wake was determined by analyzing a 1-min movement-free eyes-open segment (determined using video monitoring of NHP behavior).

The SWS quantity for each night was determined as a percentage of the recording time the subject exhibited SWS. The PSD associated with each SWS epoch was normalized by dividing the PSD by total power. The total power underlying the PSD of the respective epoch was obtained by performing trapezoidal integration of the PSD from 0.5 to 100 Hz. From the normalized PSD, the median power underlying delta (0.5–3 Hz) and beta (8–35 Hz) bands for each SWS epoch were determined. The average of SWS delta and beta power for each night was obtained for characterizing the effect of parkinsonism on cortical neural oscillations and for correlating them with SWS quantity.

### Statistical analysis

Normality was not assumed, and the Wilcoxon rank-sum (WRS) test was performed to report statistical differences. Reported *p*-values are an outcome of the WRS test unless stated otherwise. The Pearson correlation coefficient was used to assess the correlation between delta or beta power and SWS quantity. Statistical tests were performed using the statistical toolbox of MATLAB (Mathworks Inc., Natick, MA). The test results were considered significant at *p* < 0.05.

## Results

**Figure 1:**
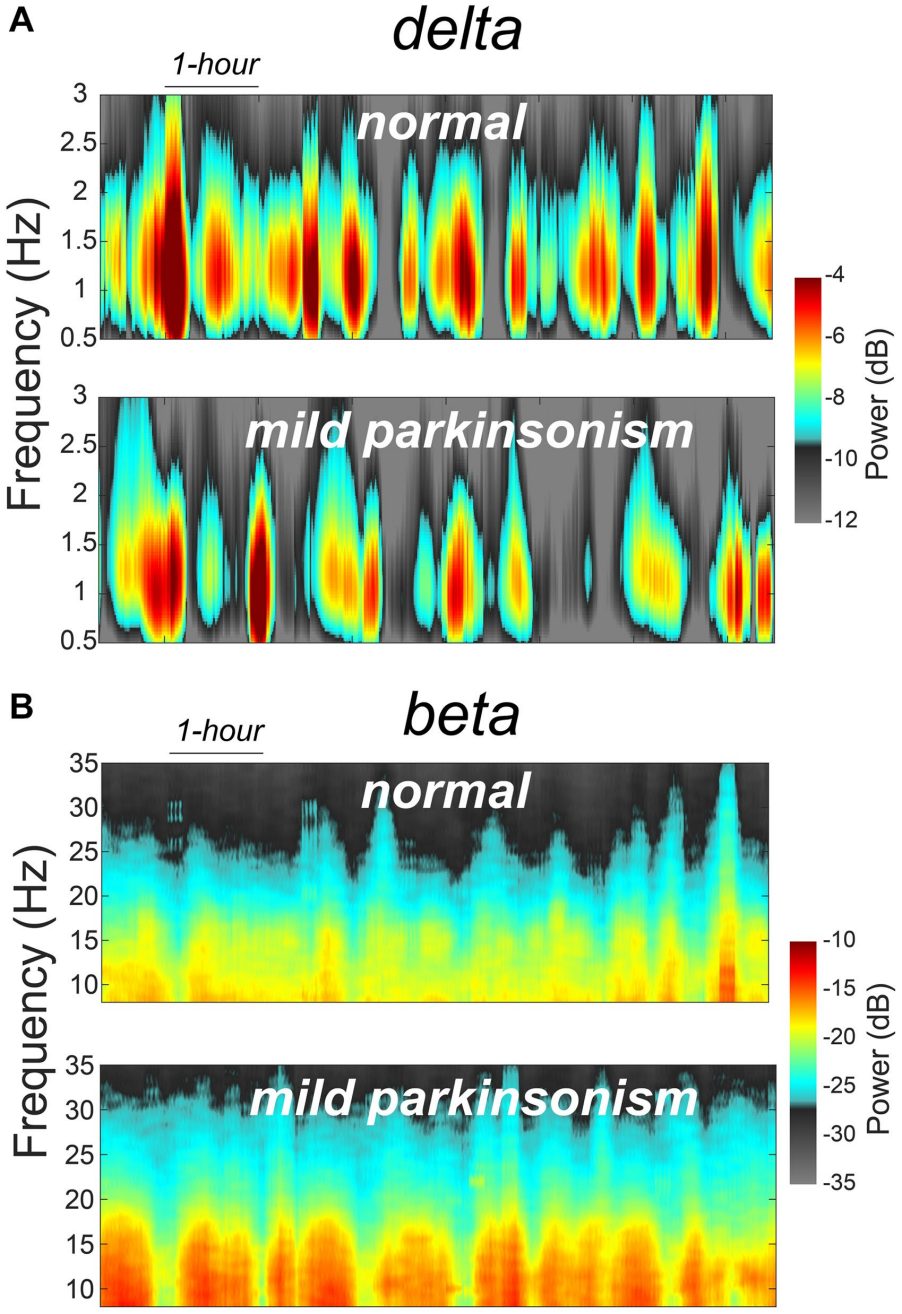
An example of MC spectrograms for one session of overnight sleep recording in normal and mildly parkinsonian states highlighting parkinsonism-related changes in delta **(A)** and beta **(B)** frequency bands.

**Figure 2:**
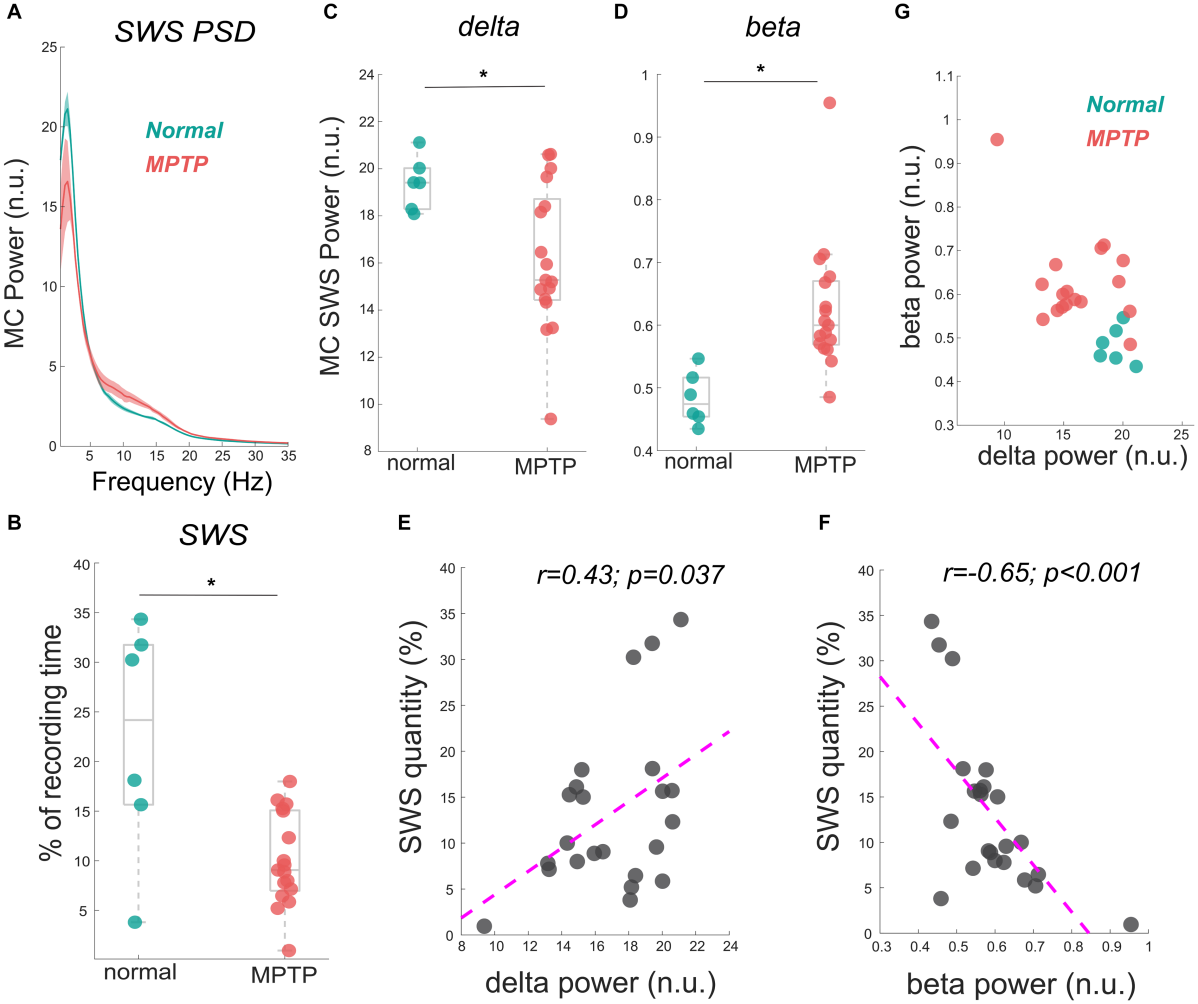
The MC PSD during SWS across normal and mild parkinsonian states shows a decrease in the delta power but an increase in beta power **(A)**. The reduction in SWS quantity (% of the recording time) in a mildly parkinsonian state compared to normal is shown in **(B)**. The distribution of delta **(C)** and beta **(D)** power across normal and parkinsonian nights shows a reduction in delta power (*p* = 0.038) but an elevation in beta power (*p* = 0.001) during SWS in a mildly parkinsonian state compared to normal. The MC delta and beta power correlated significantly with SWS quantity, this correlation is summarized (*n* = 23) in **(E,F)**, respectively. The utility of delta and beta oscillations during SWS for differentiating mild parkinsonism from the normal state is characterized in **(G)**. * Represents a significant difference between normal and MPTP (mildly parkinsonian) conditions.

The data reported in this study are from the same NHP in normal and mildly parkinsonian states. An example of overnight spectrograms highlighting the reduction in delta oscillations and elevation in beta oscillations during sleep in the mildly parkinsonian state is shown in Figures 1A,B, respectively. The spectrograms highlighting parkinsonism-associated changes across the frequency band (0.5–50 Hz) are shown in Supplementary Figure S1. The PSD (median ± median absolute deviation) across normal and parkinsonian sleep demonstrates that in the mild parkinsonian state, there was a decrease in delta and increase in beta oscillations in the MC during SWS, Figure 2A. Additionally, in mild parkinsonism we observed that SWS quantity was reduced (*p* = 0.027) compared to normal (Figure 2B). The distributions of average delta and beta power during SWS across the recording sessions in normal and parkinsonian states are shown in Figures 2C,D, respectively. The power of delta oscillations was reduced (*p* = 0.038) during SWS in the parkinsonian state compared to normal, while the power of beta oscillations was elevated (*p* = 0.001) during SWS. The median (IQR) of SWS quantity and cortical delta and beta powers during normal and parkinsonian states and the comparison (normal vs. mild parkinsonism) *p*-values for respective variables are summarized in Table 1. Furthermore, MC delta power positively correlated (*r* = 0.43; *p* = 0.037) with SWS quantity (Figure 2E), while beta power negatively correlated (*r* = −0.65; *p* < 0.001) with SWS quantity (Figure 2F), suggesting elevated beta oscillations during SWS can be detrimental for SWS. Lastly, mild parkinsonism-associated cortical power changes (delta and beta) during SWS point toward the utility of MC neural oscillations for early-stage disease classification (Figure 2G).

**Table 1:** Comparison of SWS quantity and cortical delta and beta powers during normal and mildly parkinsonian states.

Variables/conditions	Normal	Mild parkinsonism	*p*-value
SWS quantity (%)	24.17 (15.64–31.74)	9.06 (6.97–15.07)	0.027
Delta power (n.u.)	19.40 (18.28–20.02)	15.27 (14.42–18.71)	0.038
Beta power (n.u.)	0.47 (0.45–0.51)	0.59 (0.56–0.67)	0.001

The median (IQR) for each variable is presented in the table.

## Discussion

Motivated by previous studies that demonstrated the adverse effects of SWS dysfunction on the quality of life of people with advanced PD (Schreiner et al., 2021; Wood et al., 2021), we sought to understand the pathophysiology of SWS dysfunction in mild parkinsonism. The key finding of this study was that a marked reduction in SWS quantity in mild parkinsonism was accompanied by excessive beta oscillations and a reduction in delta oscillations in the MC during SWS. Our results suggest that the presence of excessive beta oscillations in the MC during SWS in mild parkinsonism has a detrimental effect on sustaining slow cortical oscillations which may contribute to the reduction in SWS quantity we observed in mild parkinsonian state.

In addition to their recognized role in the development of PD motor signs (Kuhn et al., 2008), our findings provide further support for the role of beta oscillations in the sleep dysfunction observed in patients with PD (Mizrahi-Kliger et al., 2020; Yin et al., 2023). It has recently been hypothesized that parkinsonism-related excessive beta oscillations in the basal ganglia are transmitted to the cortex, disrupting the initiation or maintenance of slow wave oscillations during sleep (Mizrahi-Kliger et al., 2020; Baumgartner et al., 2021), though the exact mechanisms by which this occurs remain unclear. Additional investigations utilizing simultaneous recordings at the single neuron and field potential level across the BGTC network will be needed to further test this hypothesis and provide key insight into the neuronal mechanisms driving the disruption of cortical delta oscillations in parkinsonian sleep and the role beta oscillations in the BGTC network may play in the emergence of sleep dysfunction in PD.

While several human and NHP studies have demonstrated that SWS is disrupted in advanced parkinsonism (Belaid et al., 2014; Mizrahi-Kliger et al., 2020; Davin et al., 2022), whether SWS dysfunction occurs during the early phase of dopaminergic system degeneration is not clear in the literature. A comparison of SWS in idiopathic REM sleep behavior disorder (RBD, often a prodromal stage of PD) patients and healthy controls has shown variable results (Massicotte-Marquez et al., 2005; Latreille et al., 2011; Rodrigues Brazète et al., 2016; Zhang et al., 2020; Sunwoo et al., 2021). A study comparing healthy controls, de-novo PD patients, and PD patients with dopaminergic treatment reported a reduction in SWS quantity in the treatment group but not in de-novo PD patients (Brunner et al., 2002). Another study found no difference in SWS quantity among controls, de-novo PD, and people with advanced PD (Amato et al., 2018). Typically, clinical sleep studies are limited to single-night polysomnography in an unfamiliar environment and lack multiple sessions of sleep recordings required to capture the variability in sleep quantity. These limitations could be a contributing factor to the variability reported in the literature as it relates to SWS dysfunction. In this regard, sleep studies using the NHP model of PD can be useful in improving pathophysiological understanding of sleep-wake dysfunction by characterizing within-subject changes with increasing disease severity.

The present study provides support for further investigation into understanding the alterations in SWS in the early stages of parkinsonism. The neural correlates of disrupted SWS (i.e., reduction in cortical delta power and elevation in beta power) that we found may translate to an early screening of PD-related disruption in SWS (Figure 2G). Furthermore, our findings provide a neural basis for the development of targeted therapies for enhancing SWS in people with early-stage PD and potentially slowing disease progression (Schreiner et al., 2019; Morawska et al., 2021). Targeted therapy in the early stage of parkinsonism may include, for example, non-invasive neuromodulation techniques (Lanza et al., 2023; Wischnewski et al., 2023) for selective amplification of delta oscillations that can enhance SWS and suppression of beta oscillations that are detrimental to SWS.

A major limitation of this study was the limited sample size. Future studies are warranted to reproduce and expand the findings reported here. The ability to perform multiple sessions of sleep recordings in the MC across normal and parkinsonian states is unique to the NHP preclinical studies and provides preliminary insights into the neural correlates of SWS dysfunction in mild parkinsonism. To further investigate the potential causal relationship between beta oscillations and disrupted SWS, future preclinical and clinical studies can employ neuromodulation techniques [e.g., DBS (Smyth et al., 2023) or noninvasive approaches like transcranial alternating current (tACS) (Johnson et al., 2020; Rossi et al., 2022)] to alter cortical beta oscillations and evaluate their effect on sleep physiology. Moreover, simultaneous neural recordings across the BGTC network and directed connectivity analysis can help decipher the mechanisms by which beta oscillations in the cortex are elevated during SWS in the early stage of parkinsonism (Mizrahi-Kliger et al., 2020; Baumgartner et al., 2021; Oswal et al., 2021). In this study, we did not study the alteration in SWS dynamics during a presymptomatic state, i.e., prior to the NHP exhibiting mild parkinsonian motor signs. This can be investigated, however, in future studies to better understand the association of alteration in the BGTC neural oscillations and SWS dynamics before the emergence of motor signs (Davin et al., 2022). Lastly, a higher number of sleep recordings across normal and early PD along with machine learning algorithms will be necessary to generalize the efficacy of features discussed in this study for early-stage disease classification.

## Data availability statement

The raw data supporting the conclusions of this article will be made available by the authors, without undue reservation.

## Ethics statement

The animal study was approved by University of Minnesota Institutional Animal Care and Use Committee. The study was conducted in accordance with the local legislation and institutional requirements.

## Author contributions

AV: Conceptualization, Formal analysis, Investigation, Methodology, Software, Validation, Visualization, Writing – original draft, Writing – review & editing. BN: Formal analysis, Investigation, Methodology, Validation, Writing – review & editing. KA: Methodology, Validation, Writing – review & editing. YY: Data curation, Writing – review & editing. EM: Data curation, Writing – review & editing. DS: Methodology, Validation, Writing – review & editing. MF: Methodology, Validation, Writing – review & editing. JW: Validation, Writing – review & editing. CM: Validation, Writing – review & editing. MH: Validation, Writing – review & editing. JV: Funding acquisition, Resources, Validation, Writing – review & editing. LJ: Conceptualization, Data curation, Formal analysis, Funding acquisition, Investigation, Methodology, Project administration, Resources, Supervision, Validation, Visualization, Writing – review & editing.
