# Supplementary material for: Slow-wave sleep dysfunction in mild parkinsonism is associated with excessive beta and reduced delta oscillations in motor cortex
Source: Front Neurosci. 2024 Feb 21;18:1338624. doi: 10.3389/fnins.2024.1338624 (PMC10915200; doi:10.3389/fnins.2024.1338624)
Supplement: Supplementary file 1 [file Data_Sheet_1.pdf]

Slow-wave sleep dysfunction in mild parkinsonism is associated with excessive beta and reduced delta oscillations in motor cortex

Ajay K. Verma[1], Bharadwaj Nandakumar[1], Kit Acedillo[1], Ying Yu[1], Ethan Marshall[1], David Schneck[2], Mark Fiecas[3], Jing Wang[1], Colum D. MacKinnon[1], Michael J. Howell[1], Jerrold L. Vitek[1], and Luke A. Johnson[1]*

[1]Department of Neurology, University of Minnesota, Minneapolis, MN, USA

[2]Masonic Institute for the Developing Brain, University of Minnesota, Minneapolis, MN, USA

[3]Division of Biostatistics, University of Minnesota, Minneapolis, MN, USA

Running Title: Role of beta oscillations in sleep dysfunction in PD

*Corresponding Author

Luke A. Johnson, PhD

Assistant Professor, Department of Neurology

Associate Director, Neuromodulation Research Center

Lions Research Building

University of Minnesota

Minneapolis, MN-55414, USA

Email: joh03032@umn.edu

Phone: 314-479-6700

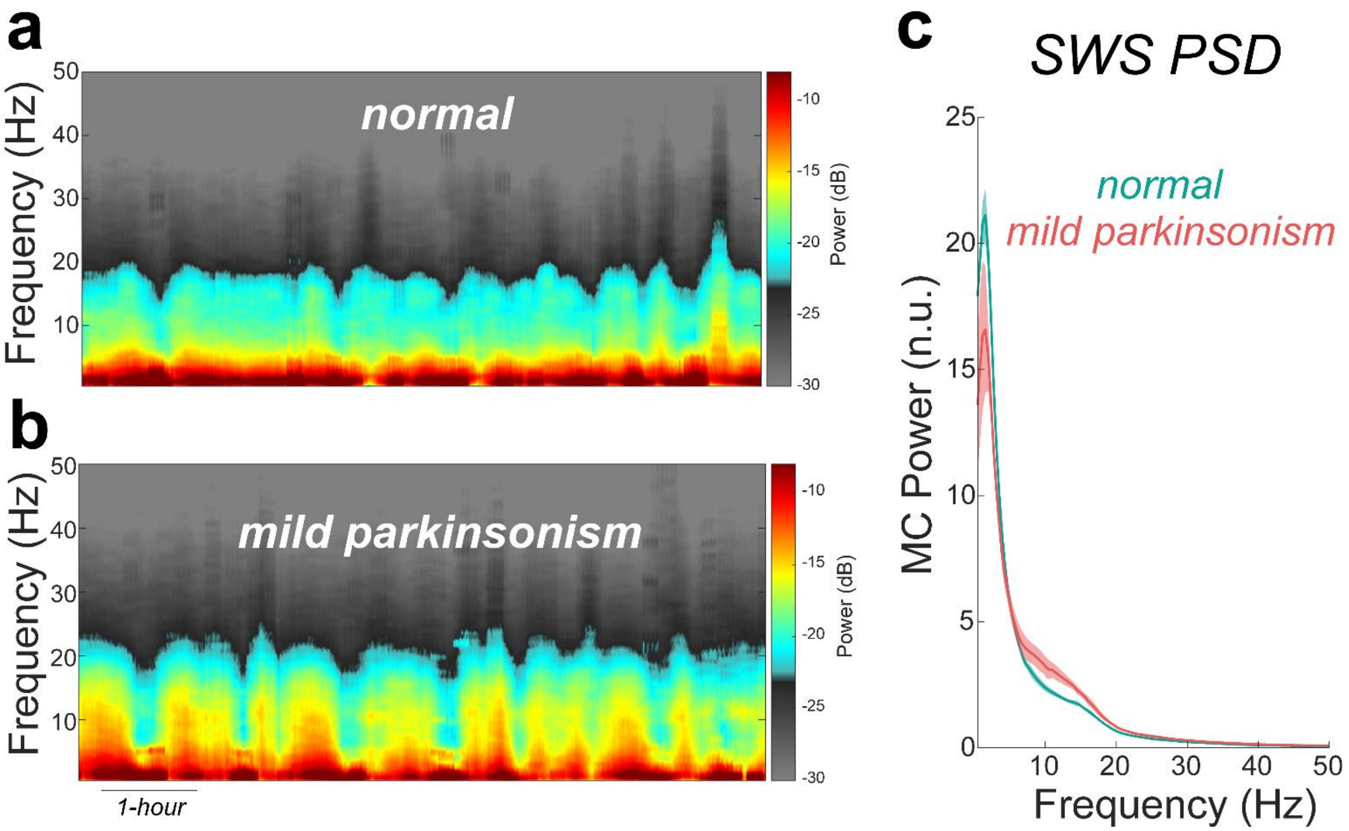

Figure S1. An example MC spectrogram (0.5-50 Hz frequency range) for one session of overnight sleep recording in normal **(a)** and mildly parkinsonian **(b)** states. Compared to normal **(a)**, excessive beta (8-35 Hz) and lower delta (0.5-3 Hz) power were noted in a mildly parkinsonian state **(b)**. The MC power spectral density during SWS across recording sessions (median±median absolute deviation) shows parkinsonism associated changes in 0.5-50 Hz frequency range **(c)**.
